# Discordance between Body-Mass Index and Body Adiposity Index in the Classification of Weight Status of Elderly Patients with Stable Coronary Artery Disease

**DOI:** 10.3390/jcm10050943

**Published:** 2021-03-01

**Authors:** Bartosz Hudzik, Justyna Nowak, Janusz Szkodzinski, Aleksander Danikiewicz, Ilona Korzonek-Szlacheta, Barbara Zubelewicz-Szkodzińska

**Affiliations:** 1 Department of Cardiovascular Disease Prevention, Department of Metabolic Disease Prevention, Faculty of Health Sciences, Medical University of Silesia, 41-900 Bytom, Poland; justyna.nowak@sum.edu.pl (J.N.); ikorzonek@sum.edu.pl (I.K.-S.); 2 Third Department of Cardiology, Silesian Center for Heart Disease, Faculty of Medical Sciences, Medical University of Silesia, 41-800 Zabrze, Poland; janszkod@poczta.onet.pl; 3 Department of Nutrition-Related Disease Prevention, Department of Metabolic Disease Prevention, Faculty of Health Sciences, Medical University of Silesia, 41-900 Bytom, Poland; adanikiewicz@sum.edu.pl (A.D.); basiazub@poczta.onet.pl (B.Z.-S.); 4 Department of Endocrinology, District Hospital, 41-940 Piekary Śląskie, Poland

**Keywords:** body-mass index, body adiposity index, weight status classification, discordance, coronary artery disease, elderly

## Abstract

Background and Aims: Body-mass index (BMI) is a popular method implemented to define weight status. However, describing obesity by BMI may result in inaccurate assessment of adiposity. The Body Adiposity Index (BAI) is intended to be a directly validated method of estimating body fat percentage. We set out to compare body weight status assessment by BMI and BAI in a cohort of elderly patients with stable coronary artery disease (CAD). Methods: A total of 169 patients with stable CAD were enrolled in an out-patient cardiology clinic. The National Research Council (US) Committee on Diet and Health classification was used for individuals older than 65 years as underweight BMI < 24 kg/m^2^, normal weight BMI 24–29 kg/m^2^, overweight BMI 29–35 kg/m^2^, and obesity BMI > 35 kg/m^2^. In case of BAI, we used sex- and age-specific classification of weight status. In addition, body fat was estimated by bioelectrical impedance analysis (BImpA). Results: Only 72 out of 169 patients (42.6%) had concordant classification of weight status by both BMI and BAI. The majority of the patients had their weight status either underestimated or overestimated. There were strong positive correlations between BMI and BImpA (FAT%) (R = 0.78 *p* < 0.001); BAI and BImpA (FAT%) (R = 0.79 *p* < 0.001); and BMI and BAI (R = 0.67 *p* < 0.001). BMI tended to overestimate the rate of underweight, normal weight or overweight, meanwhile underestimating the rate of obesity. Third, BMI exhibited an average positive bias of 14.4% compared to the reference method (BImpA), whereas BAI exhibited an average negative bias of −8.3% compared to the reference method (BImpA). Multivariate logistic regression identified independent predictors of discordance in assessing weight status by BMI and BAI: BImpA (FAT%) odds ratio (OR) 1.29, total body water (%) OR 1.61, fat mass index OR 2.62, and Controlling Nutritional Status (CONUT) score OR 1.25. Conclusions: There is substantial rate of misclassification of weight status between BMI and BAI. These findings have significant implications for clinical practice as the boundary between health and disease in malnutrition is crucial to accurately define criteria for intervention. Perhaps BMI cut-offs for classifying weight status in the elderly should be revisited.

## 1. Introduction

Overweight and obesity are among major risk factors for cardiovascular disease (CVD) and are closely linked to morbidity and mortality worldwide [1]. Obesity is associated with increased atherosclerotic diseases, especially coronary artery disease (CAD) via numerous mechanisms [2,3,4]. The authors of the Framingham Heart Study reported that, compared with normal weight, being overweight was associated with a shorter life expectance by three years, whereas obesity was linked to a 6–7-year decrease in life expectancy. Obesity also increased the risk for premature death in men by 81% and by 115% in women [5]. Notwithstanding, despite evidence of a positive correlation between high adiposity and cardiovascular morbidity and mortality, there is emerging evidence for better clinical outcome in patients with overweight or obesity. This phenomenon was coined the obesity paradox [6,7].

Body-mass index (BMI) is a popular and easy-to-use method which is regarded to as the standard modality in defining weight status. However, BMI is merely a measure of weight adjusted for height and, thus, should be only interpreted as a surrogate parameter in the assessment of adiposity. Although BMI is ideal for epidemiological studies, assessment only by BMI can result in inaccurate estimation of adiposity, because the numerator in the BMI formula does not distinguish lean body mass from fat mass [8]. Dual-energy X-ray absorptiometry (DXA) allows for a fast and non-invasive assessment of fat mass (FM) and fat-free mass (FFM). Thus, it is considered to be the gold standard (reference method) in clinical research. The major shortcoming of DXA is that it requires specialized radiology equipment. Moreover, it is expensive. All of these factors make DXA unfeasible in routine clinical practice [9]. Studies that compared adiposity (body fat rates) evaluated by DXA and other modalities with BMI reported a substantial rate of discordance and misclassification of adiposity status by BMI in many conditions [10], including stable CAD [11,12]. In addition, there are numerous aspects which make the BMI a suboptimal measure of adiposity in the elderly. First and foremost, the loss of height specific for aging which is the result of vertebral body compression and angulation of the spine leads to the overestimation of adiposity by BMI. In addition, BMI does not account for fat distribution. Hence, the use of BMI for assessing adiposity in the elderly has been largely criticized in view of the fact that it does not account for age-related changes in adipose tissue—more specifically that it does not allow for the evaluation of the ratio between FM and FFM [13].

The Body Adiposity Index (BAI) has been calculated from hip circumference and height. BAI was proposed to be a validated method of estimating body fat percentage (BF%). The final formula was shown to predict percentage of body adiposity assessed by DXA. Thus, BAI was said to overcome the shortcomings of the BMI in discriminating between fat and lean mass [14,15,16].

Obesity is the underlying cause of many cardiovascular diseases—the foremost physical consequence of obesity is atherosclerotic cardiovascular disease (ASCVD) [8,17]. In patients with CAD, BMI does not discriminate between BF% and lean mass, and a BMI < 30 kg/m^2^ is a poor index with which to diagnose obesity. These findings may explain the controversial findings that link mild elevations of BMI to better survival and fewer cardiovascular events in patients with CAD. Techniques to accurately define obesity in patients with CAD might be necessary [11]. In addition, adiposity status may also affect the efficacy of cardiovascular therapies. A meta-analysis of 29 randomized controlled trials of the relation of body mass index to cardiovascular outcomes in patients receiving intensive low-density lipoprotein cholesterol (LDL-C) lowering therapy demonstrated that patients with normal BMI treated with intensive LDL-C lowering regimens may derive a larger clinical benefit compared with patients with larger BMI [18].

The ambiguities about the appropriate level of obesity that should trigger an intervention, due to age-related physiologic changes and a lack of consensus on specific criteria and cutoffs remain an important barrier. When assessing older adults on an individual basis, especially with regard to implementing weight reduction strategies, the alternatives to BMI deserve serious consideration in light of their implications for health, preventative or otherwise. The comparison of weight status assessment by BMI versus BAI has been poorly documented particularly among older adults with stable CAD. Few studies have pointed out good concordance between the two modalities while other have not. In our study, we set out to compare body weight status assessment by BMI and BAI in a cohort of elderly patients with stable CAD.

## 2. Materials and Methods

The study was approved by the bioethics board at the Medical University of Silesia. It conforms to the Declaration of Helsinki (KNW/0022/KB1/53/14). A total of 169 patients with stable CAD were enrolled. Stable CAD was defined as a history of documented myocardial infarction, prior coronary revascularization, or chest pain with documented myocardial ischemia. Given the reported significant effect of fluid accumulation on weight and bioelectric impedance measurements and to assure the evaluation of edema-free mass, the patients with decompensated heart failure and uncontrolled hypertension were excluded from the study. The exclusion criteria were pregnancy or lactation or implanted electrical devices. Detailed inclusion and exclusion criteria were previously published [19].

### 2.1. Anthropometric Measurements

Body weight was measured to the nearest 0.05 kg, using a calibrated scale (B150L, Radwag, Radom, Poland). Height was measured to the nearest 0.1 cm, using a stadiometer (SECA 217). Hip circumference (HC) was measured at the level of the greater trochanter of the femoral bone. Waist circumference (WC) was measured at the smallest circumference between the costal margin and the iliac crest. Waist-to-hip ratio (WHR) was calculated as WC divided by HC. Waist-to-height ratio (WHtR) was calculated as WC divided by height.

BMI was calculated by dividing weights in kilograms by height in meters squared. Owing to much criticism for the use of BMI in elderly populations considering it does not take into account age-associated changes, we used the National Research Council (US) Committee on Diet and Health classification of weight status in persons 65 years or older as underweight—BMI < 24 kg/m^2^, normal weight—BMI 24–29 kg/m^2^, overweight BMI—29–35 kg/m^2^, and obesity—BMI > 35 kg/m^2^ [20].

BAI was calculated according to the following formula: [14]

(1) BAI = Hip circumference [cm](Height [m])^1.5 − 18,

In case of BAI, we used sex- and age-specific classification of weight status (Table 1) [21].

### 2.2. Bioimpedance Analysis (BImpA)

Body fat was evaluated by bioelectrical impedance analysis (BImpA) using a Tanita instrument (TBF-300A). FM was estimated by BImpA. BImpA-derived body fat percentage (BF%) equations were used to estimate lean body mass (LBM), FFM, total body water (TBW) and body fat mass. We used the following conversions to estimate BF%: FFM = 0.97 × LBM for men and FFM = 0.92 × LBM for women; FFM = TBW/0.73; BF% = (body weight − FFM)/body weight [22,23]. The calculations of fat-free mass were made based on the Kyle formula [24].

The Controlling Nutritional Status (CONUT) score was developed as a screening tool to identify malnourished patients or patients at risk for malnutrition [25]. It is derived from serum albumin, total lymphocyte count, and total cholesterol. A higher score means worse nutritional status.

### 2.3. Statistical Analysis

Quantitative data are presented as medians and interquartile ranges (lower and upper quartiles). Qualitative data are presented as frequencies. Kruskal–Wallis ANOVA was used to test the differences between the three groups. The relationship between BMI and BAI was evaluated by Spearman’s rank correlation coefficient. All variables with a ”*p*” value of less than 0.05 in the univariate analysis entered into the multivariate logistic regression model using the Wald statistic backward stepwise selection. Multivariate logistic regression analysis was employed to evaluate odds ratios (OR) and 95% confidence intervals (95% CI) to identify independent factors associated with BMI–BAI discordance in categorizing weight status. The coefficient of determination (R^2^) and standard error of the estimate (SEE), which is the standard deviation of the data points around the regression line, were calculated. Bland–Altman plots were also used to compare BAI and BMI with standard body fat measures. A range of agreement was based on the mean bias, or mean difference between the means of the measures ± 2 SD, an interval within which 95% of the differences between BAI/BMI indices and the more direct % fat measurements (i.e., BImpA). A receiver operating characteristic (ROC) analysis was performed to assess the predictive value in identifying the discrimination thresholds of BMI for all BAI-defined weight categories and predictive value in identifying the discrimination thresholds of BAI for all BMI-defined weight categories. A value of *p* < 0.05 was considered significant.

## 3. Results

**Figure 1:**
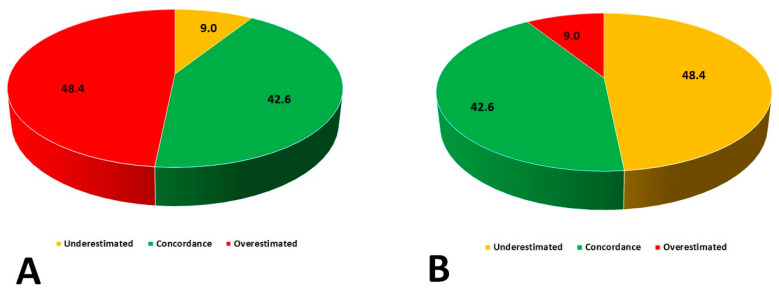
Differences in weight status classification based on BMI and Body Adiposity Index (BAI). (**A**) Concordance of assessing adiposity status by BAI (Reference BMI); (**B**) Concordance of assessing adiposity status by BMI (Reference BAI).

**Figure 2:**
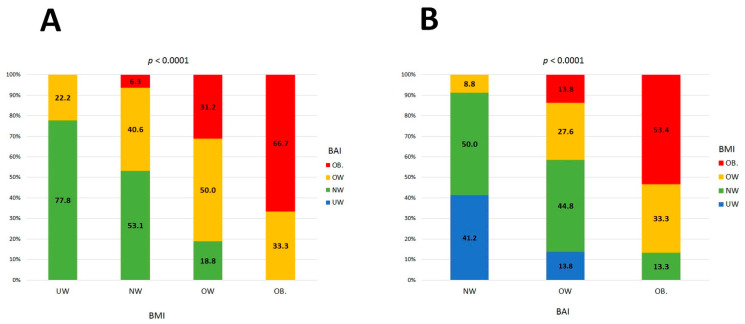
(**A**) Discordance between classification of weight status categories by BAI (BMI reference); (**B**) discordance between classification of weight status categories by BMI (BAI reference).

Baseline clinical and laboratory characteristics are presented in Table 2. All enrolled patients were Caucasian and the majority of study participants were women. A high rate of hypertension and heart failure was observed. There were substantial differences in weight status classification based on BMI and BAI (Table 3, Figure 1). Only 72 out of 169 patients (42.6%) had concordant classification of weight status by both BMI and BAI. More than half of the patients had their weight status either underestimated or overestimated. There were no patients classified as underweight when using BAI as a reference method. Numerous discrepancies were seen across all weight status by BAI with respect to BMI categories (Figure 2). There were strong positive correlations between BMI and BImpA (%BF) (R = 0.78 *p* < 0.001); BAI and BImpA (FAT%) (R = 0.79 *p* < 0.001); and BMI and BAI (R = 0.67 *p* < 0.001).

**Figure 3:**
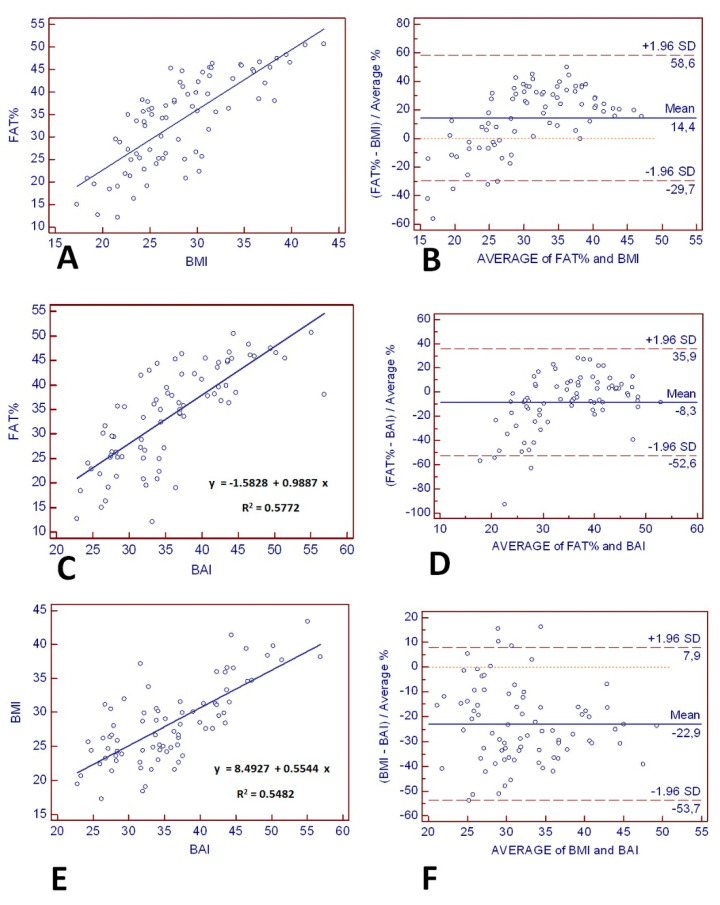
The line of regression for (**A**) bioelectrical impedance analysis (BImpA) (FAT%) and BMI, (**C**) BImpA (FAT%) and BAI, and (**E**) BMI and BAI is represented by the solid line, and the line of equality by the dotted line. The Bland–Altman Plot (right panels) provides an interval within which 95% of differences between derived from (**B**) BMI–BImpA (FAT%), (**D**) BAI–BImpA (FAT%), and (**F**) BMI–BAI. The thick black line represents the mean bias (mean of the differences), the dotted line (line of equality), and the two thin horizontal lines are drawn at the 95% confidence interval of limits of agreement (mean bias ± 2SD).

The regression lines and lines of equality assessed the correlation and agreement between BAI and BMI with % fat estimates from BImpA. Figure 3A,C,E show that the variation (the standard error of estimate (SEE)) around the regression lines was similar for BMI and BAI comparisons with BImpA (BMI with BImpA: SEE = 6.40 and BAI with BImpA: SEE = 6.54) and for BMI comparison with BAI (SEE = 3.90). The 95% limits of individual agreement between BMI and BImpA (FAT%) were −29.7% to 58.6% (range: 88.3%), as shown in Figure 3B. These limits of individual agreement were similar to those found between BAI and BImpA (FAT%) (−52.6% to 35.9%, range: 88.5%), as shown in Figure 3D. However, BMI exhibited an average positive bias of 14.4% compared to the reference method, whereas BAI exhibited an average negative bias of −8.3% compared to the reference method. Figure 3F depicts the Bland–Altman plot comparing weight status estimated by the BAI and BMI. The limits of agreement (95% confidence intervals) between the BAI and BMI ranged between −53.7% and 9.9%. BAI exhibited an average negative bias of −22.9% compared to BMI.

Multivariate logistic regression identified independent predictors of discordance in assessing weight status by BMI and BAI: BImpA (%BF) OR 1.29 (95%CI 1.18–1.43) *p* = 0.02, total body water (%) OR 1.61 (95%CI 1.09–2.32) *p* = 0.04, fat mass index OR 2.62 (95%CI 1.23–5.60) *p* < 0.001, and CONUT score OR 1.25 (95%CI 1.01–1.69) *p* = 0.04. 

ROC analysis demonstrated a high predictive value in identifying the discrimination thresholds of BMI for all BAI-defined weight categories. When speaking of BMI-defined weight categories, ROC analysis demonstrated a high predictive value in identifying the discrimination thresholds of BAI only for underweight and obesity (a wide variety of results across normal weight and overweight defined by BMI) (Table 4).

## 4. Discussion

In the current study, we set out to compare body weight status which has been assessed by BMI in comparison to BAI in a cohort of elderly patients with stable CAD. There are several key findings of this study. First and foremost, there is a substantial rate (over 50%) of misclassification of weight status between BMI and BAI. Second, the accuracy of BMI in predicting adiposity status assessed by BAI is fairly poor in elderly patients with stable CAD. BMI tended to overestimate the rate of underweight, normal weight or overweight, meanwhile underestimating the rate of obesity. Third, BMI exhibited an average positive bias of 14.4% compared to the reference method (BImpA), whereas BAI exhibited an average negative bias of −8.3% compared to the reference method (BImpA). Fourth, BAI exhibited an average negative bias of −22.9% compared to BMI. Finally, independent predictors of discordance in classifying weight categories by BMI and BAI were: body fat (assessed by BImpA), total body water, fat mass index, and poor nutritional status (assessed by CONUT score).

BMI is a measure of weight adjusted for height and is commonly viewed as a marker of body fatness. However, it is hardly a surrogate measure of adiposity because it measures excess weight rather than excess fat. Furthermore, BMI does not account for the distribution of body fat [26]. In the elderly, the use of BMI to classify weight status brings yet another set of issues including, but not limited to the stature decline, reductions in LBM, accumulation of fatty tissue, and decrease in the amount of body fluids. For these reasons, the use of BMI in assessing overweight and obesity in the elderly may be dubious, despite the age- and sex-specific cut-offs. Batsis et al. studied 4984 subjects aged 60 years or older [27]. A BMI of 30 or higher had a low sensitivity and moderate specificity (for men 32.9% and 80.8%, for women 38.5% and 78.5%) which resulted in a correct classification of 41% and 45% of obese individuals. A BMI of 25 or higher had a moderate sensitivity and specificity (for men 80.7% and 99.6%; for women 76.9% and 98.8%,) which resulted in a correct classification of 80% and 78% of obese individuals. Authors came to the conclusion that BMI may be a suboptimal marker for adiposity in the elderly [27].

DXA has become a gold standard for estimating body composition, providing data on FM and FFM. The use of DXA, however, is expensive and not widely available [8,28]. Therefore, the use of more accessible modalities for measuring adiposity would have important practical applications. In an attempt to enhance the modalities commonly used to estimate %BF, Bergman et al. suggested BAI, which demonstrated consistent association (r = 0.85) with fat percentage measured by DXA [14]. BAI does not require the use of complex and costly imaging methods or DXA. In addition, it offers advantages in comparison to other less expensive tools for estimation of %BF, such as measurement of skinfold thickness or BImpA, which have been criticized as being inaccurate. Studies have found that BIA becomes less accurate as BF increases. In addition, the accuracy of BIA largely depends on the equation used. BIA measurements validated for specific ethnic groups, populations and conditions can accurately measure body fat in those populations [29].

Barreria et al. reported that correlations with %BF and fat mass were similar for BMI and BAI, thus stating that BMI and BAI perform similarly in predicting %BF [30]. Notwithstanding, several other studies evaluated BAI as a predictor of BF composition, yielding conflicting results. Bennasar-Veny et al. studied Caucasians and reported that adiposity indices that included the waist circumference (WHtR and WC) may be better tools than BAI or BMI in the evaluation of metabolic and cardiovascular risk in both everyday clinical practice and research [31]. Conversely, Johnson et al. who investigated 623 European-American adults reported that BAI provided a better estimation of adiposity status than did BMI. However, it did not provide valid estimates of %BF, particularly in individuals with lower levels of body fatness [32].

Notably, validation studies performed in populations of patients of various ethnicities have consistently suggested that the BAI tends to overestimate adiposity at lower BF%, and underestimate adiposity at higher BF% [33,34,35]. Therefore, it would seem that BAI is not an optimal tool for estimating adiposity in specific populations, notably in patients of Asian descent in whom discriminatory capacity of the BMI is higher than the one of BAI [36]. Zhang et al. demonstrated that BAI compared with BMI and waist circumference was neither a better predictor for BF% nor for cardiovascular risks in the Chinese population [37]. Noteworthy, compared with Caucasian patients, all racial/ethnic minority groups (Chinese Americans, African Americans, Hispanics, and South Asians) had a significantly higher prevalence of metabolic abnormality but normal weight, which was not explained by demographic, behavioral, or ectopic fat measures [38].

In our study, we demonstrated that BAI presented a better agreement with BImpA than did BMI. Bland–Altman plots provided wider 95% confidence intervals for BMI difference comparisons than they do for BAI difference comparisons for their respective means for BImpA. Moreover, BMI and BAI showed similar strong correlations with BImpA; the correlation between BAI and BMI was only moderate. Less than half of the study participants had concordant classification of weight status by both BMI and BAI. The majority of patients had their weight status either underestimated or overestimated. Independent predictors for misclassification of weight status included indicators of increased weight (body fat percentage, total body water percentage, fat mass index) and decreased weight (CONUT).

## 5. Conclusions

In summary, we identified a high rate of misclassification of weight status between BMI and BAI. These findings have important clinical ramifications for everyday practice as the line between health and disease in malnutrition (overnutrition and undernutrition alike) in terms of body composition is significant for an accurate definition of the standards for intervention, notably the methods and intensity of nutritional intervention. Notwithstanding, this area of research still represents a clinical challenge that needs to be addressed. This study raised important questions about using BMI to define the degree of obesity in older adults with stable CAD that is necessary to define the intensity of the clinical interventions (nutritional, pharmacological, psychological, rehabilitation, and surgical). More importantly, perhaps BMI cut-offs for classifying weight status in the elderly should be revisited.

## 6. Study Limitations

Our study needs to be viewed in light of its limitations. The relatively small number of patients in our cohort could have rendered some differences insignificant, and, thus, this subject needs further examination in larger cohorts. The validity of BAI was not evaluated in the Polish population. Likewise, future studies should include comparing BAI to DXA measurements, which are considered the gold standard. In addition, specifically in the elderly, given the aforementioned age-related changes in body composition, it is also important to consider the criterion methods used to calibrate the predictive equations. Age-specific body composition changes have a substantial impact on the BImpA measurements. As such, the predictive equations are obtained from linear regression analysis, and thus the use of cross-validated equations developed in populations with similar biological and clinical characteristics is essential in order to guarantee both the accuracy and the precision of the estimates when applying these equations in other populations. The calculations of fat-free mass were made based on the Kyle formula [24]. Although not being age-specific, it can be used in people up to the age of 94 years and is one of several cross-validated BImpA equations for the elderly, which were calibrated against DXA as a criterion method [39].

## Figures and Tables

**Table 1:** Age- and sex-specific cut-offs for assessing weight status by body adiposity index (BAI).

Weight Status	Age ≥ 60 Years	
	Men	Women
Underweight	<13%	<25%
Normal weight	13–25%	25–38%
Overweight	26–31%	39–43%
Obesity	>31%	>43%

**Table 2:** Baseline clinical and laboratory characteristics.

Sex, men *n* (%)	52 (30.8%)
Age (years)	75 (70–84)
Prior myocardial infarction *n* (%)	30 (18.0)
Heart failure *n* (%)	73 (43.2%)
Atrial fibrillation *n* (%)	28 (16.7)
Hyperlipidemia *n* (%)	39 (23.1)
Diabetes mellitus *n* (%)	63 (37.3)
Hypertension *n* (%)	154 (91.1)
Leucocytes (10^3^/mm^3^)	6.5 (5.2–8.4)
Erythrocytes (10^6^/mm^3^)	4.2 (3.9–4.5)
Hemoglobin (g/dL)	12.5 (12.7–13.4)
Hematocrit (%)	38 (36–40)
Platelets (10^3^/mm^3^)	215 (171–262)
Total cholesterol (mmol/L)	4.4 (3.6–5.3)
HDL cholesterol (mmol/L)	1.3 (1.1–1.7)
LDL cholesterol (mmol/L)	2.6 (1.7–3.2)
Triglycerides (mmol/L)	1.1 (0.9–1.4)
Serum creatinine (μmol/L)	75 (65–91)
eGFR (mL/min/1.73 m^2^)	76 (61–89)
Aspartate aminotransferase (AST) (U/L)	18 (15–21)
Alanine aminotransferase (ALT) (U/L)	15 (11–20)
Bilirubin (mmol/L)	10.7 (8.5–14.7)
Total protein (g/L)	60.0 (56.2–65.3)
Albumin (mg/mL)	36 (34–40)
CONUT score	2 (1–3)

CONUT score—Controlling Nutritional Status Continuous score; variables are given as medians and interquartile ranges; eGFR—estimated glomerular filtration rate; HDL—high-density lipoprotein; LDL—low-density lipoprotein.

**Table 3:** Anthropometric and body composition measurements.

Weight (kg)	70 (61–79)
Height (m)	1.68 (1.58–1.75)
BMI	27.6 (24.3–31.3)
BAI	34.6 (29.0–42.1)
Weight status by BMI	
Underweight, *n* (%)	39 (23.1)
Normal weight, *n* (%)	69 (41.0)
Overweight, *n* (%)	35 (20.7)
Obesity, *n* (%)	26 (15.2)
Weight status by BAI	
Underweight, *n* (%)	0 (0)
Normal weight, *n* (%)	74 (43.8)
Overweight, *n* (%)	63 (37.3)
Obesity, *n* (%)	32 (18.9)
Concordant classification of weight status by both BMI and BAI, *n* (%)	72 (42.6)
Hip circumference (cm)	104 (97–112)
Waist circumference (cm)	103 (90–114)
Mid-upper arm circumference (cm)	28 (26–31)
Calf circumference (cm)	34 (32–37)
Waist-to-hip ratio	0.97 (0.90–1.00)
Waist-to-height ratio	0.64 (0.56–0.73)
Fat percentage (%)	33.6 (26.3–42.3)
Fat mass (kg)	22.8 (16.3–32.6)
Lean mass (kg)	42.8 (38.9–61.7)
Muscle mass (kg)	40.6 (36.9–49.1)
Total body water percentage (%)	29.7 (26.2–37.0)
Total body water percentage (kg)	43.8 (39.6–50.7)

Continuous variables are given as medians and interquartile ranges.

**Table 4:** Receiver-operating characteristics (ROC) curves identifying the discrimination thresholds of BAI and BMI for each weight status categories set out by BAI and BMI.

BAI Weight Status							
	BMI cutoff	AUC (95%CI)	Sensitivity (%)	Specificity (%)	PPV (%)	NPV (%)	*p*
Normal weight	≤27	0.88 (0.78–0.94)	85	79	76	87	<0.0001
Overweight	>25.7	0.61 (0.51–0.72)	83	51	50	83	0.05
Obesity	>28.7	0.93 (0.85–0.97)	100	71	45	100	<0.0001
**BMI weight status**							
	BAI cutoff	AUC (95%CI)	Sensitivity (%)	Specificity (%)	PPV (%)	NPV (%)	*p*
Underweight	<33.1	0.78 (0.68–0.87)	78	72	45	91	<0.0001
Normal weight	<43.5	0.60 (0.48–0.70)	100	28	49	100	0.13
Overweight	>37	0.62 (0.50–0.73)	62	69	34	88	0.11
Obesity	>42	0.92 (0,82–0,97)	92	88	61	98	<0.0001

AUC—are under the curve; BAI—body adiposity index; BMI—body-mass index; CI—confidence interval; NPV—negative predictive value; PPV—positive predictive value; ROC—receiver-operating characteristics.

## Data Availability

The data presented in this study are available on request from the corresponding author. The data are not publicly available due to restrictions that apply to the availability of these data.
